# Comparison of Effectiveness of Topiramate and Diazepam in Preventing Risk of Recurrent Febrile Seizure in Children under Age of 2 Years

**Published:** 2018

**Authors:** Afshin FAYYAZI, Ali KHAJEH, Ashraf BAGHBANI

**Affiliations:** 1 Department of Pediatric Neurology, Hamadan University of Medical Sciences, Hamadan, Iran; 2 Department of Pediatric Neurology, Zahedan University of Medical Sciences, Zahedan, Iran

**Keywords:** Febrile seizure, Prevention, Diazepam, Topiramate

## Abstract

**Objective:**

Febrile seizures are the most common type of convulsions. Medicinal prophylaxis is sometimes used for children at high risk of recurrent febrile seizure. In certain circumstances, conventional drugs such as diazepam and phenobarbital cannot be used and the need for alternative medicines is felt. This study compared the effectiveness of topiramate and diazepam in preventing the risk of recurrent febrile seizure in children under 2 yr old.

**Materials and Methods:**

This randomized controlled trial, in Besat Hospital in Hamedan, Iran from 22 Nov 2013 to 22 Nov 2015 (Registered code: IRCT Number: IRCT2015010120527N1), included 54 patients, at risk of recurrent febrile seizure, inhibited from taking phenobarbital. Samples were randomly divided into two groups. The first group received diazepam treatment during fever episodes and the second group received daily dose of topiramate. A one-year follow-up of recurrent febrile seizure and its complications was also conducted.

**Results:**

Thirty-four patients (17 patients in each group) completed the one-year course of the trial. Recurrent febrile seizure was not observed in the course of preventive treatment. The prevalence of minor complications was 29.4% in the diazepam group and 48.5% in the topiramate group. No major complication was observed in among the subjects

**Conclusion:**

Topiramate can be recommended for preventing recurrent febrile seizure when the use of frontline medicines is not possible.

## Introduction

Febrile seizure is the most common type of convulsion in childhood with the prevalence of 2% - 5% among children between the ages of 6 and 60 months (1-3). The prevalence of febrile seizure is higher in some regions (e.g. 9%-10% in Japan versus 14% in the Mariana Islands in Guam) (2). About 21% of seizure attacks happen before or within 1 h after the onset of fever, and only 22% occur more than 24 h after the onset of fever (2). 

Almost one-third of all children with febrile seizures will have a recurrence. Age is the main risk factor of the recurrence with 50% probability under the age of 1 year. The first recurrence after an initial febrile seizure usually occurs within a year of the initial seizure. A first-degree family history positive for febrile seizures increases the risk of a recurrence. In addition, children who have an initial seizure with a low-grade fever are more prone to a recurrence (3, 4). Simple febrile seizures are a benign phenomenon not associated with increased rate of mortality, and cognitive and cerebral complications (3, 5). 

The risk of epilepsy in people at 25 yr with a history of simple febrile seizure after age 1 year is as high as normal people (1%). The risk of developing epilepsy in children with an initial febrile seizure before age 1 year and with family history positive for epilepsy is 24% at age 25 (3). On the other hand, scant evidence of cerebral traumas has been observed among patients with a history of a prolonged febrile seizure. According to magnetic resonance imaging examinations of the brain, acute brain changes have been observed in 30%-40% of children with a prolonged febrile seizure (90-100 min) (5). Forty percent of people with mesial temporal epilepsy had a history of prolonged seizures in childhood (2).

 Concerning the benign nature of febrile seizures in most cases, the main goal of treating such patients is to prevent the incidence of a prolonged seizure. To this end, diazepam is given rectally at home or during transfer to hospital; in addition, anticonvulsant medications are used in hospital for seizure management (2). Sometimes a preventive treatment is given to a child with a febrile seizure, owing to the incidence of prolonged seizures, parental anxiety, and the hard-to-access medical centers (6). There are currently 3 major recurrent febrile seizure prevention techniques: (i) daily dose of oral phenobarbital, (ii) daily dose of oral sodium valproate, and (iii) oral diazepam during fever episodes. Each of these methods has its own strengths and weaknesses (7-9).

 Phenobarbital is an effective medicine for the prevention of a recurrent febrile seizure when its serum concentration reaches15 µg/mL. The intake of phenobarbital has decreased yearly incidence of 25 types of seizures to five (3, 10). However, such complications as allergic reactions, as well as cognitive and behavioral disorders are widely observed in these patients (20%-40%) (3). On a yearly average, the use of diazepam during fever episodes decreases the incidence risk of febrile seizure by 44% (ibid). However, the administration of diazepam is challenging for parents because they have to measure the child's body temperature regularly. In addition, there is a risk of an anti-fever seizure (21%). Moreover, diazepam may be associated with such complications as restlessness, balance disorders, irritability and drowsiness (10-12). Daily dose of sodium valproate is effective in febrile seizure management, with 4% risk for recurrence versus 35% in control group per year (3). In some circumstances, physicians decide to administer prophylactic therapy for a child having febrile seizure, but the use of conventional medicines is not possible. For example, phenobarbital-induced allergy and recurrent febrile seizure in early hours after the onset of fever in a neonate inhibit prophylaxis with benzodiazepines. As a result, the need for an alternative medicine is felt. 

Some anticonvulsants (e.g. carbamazepine and phenytoin are) are inefficient in febrile seizure prevention (13-15). There are few studies on the effectiveness of newer anticonvulsants (2). Some scant evidences represent the probable effectiveness of topiramate in febrile seizure management (16-18).

This drug is widely used for controlling partial and generalized seizures, shown effective in neonatal seizure control, applicable in young ages, and proven to be effective in seizure syndromes with genetic causes such as febrile seizure and Dravet syndrome (2). 

This study was set out to compare the effects and side effects of topiramate and diazepam administered to prevent the risk of recurrent febrile seizure in children before the age two years.

## Materials and Methods

This is a non-blinded randomized controlled trial performed in Besat Hospital in Hamedan, Iran (Registered code: IRCT Number: IRCT2015010120527N1). Cases were selected by convenience sampling from 22 Nov 2013 to 22 Nov 2015. Ethics Committee of Hamadan University of Medical Sciences approved the study.

This study was conducted on 54 children aged 1 month to 2 yr, who were on preventive treatment with phenobarbital for high risk of recurrent febrile seizure, but discontinued the medications after developing drug-induced complications.

Eligible criteria for receiving preventive drugs were as follows:

Recurrent febrile convulsion.

Status epilepticus (a seizure lasting more than 30 min or two successive seizures lasting more than 30 min with no recovery between them).

Febrile seizure along with at least two risk factors for recurrence: seizure within the first 24-h after the onset of fever, seizure at body temperature below 39 °C, age before 12 months, family history of fever, and complex febrile seizure.

Living in hard-to-reach regions remote from medical centers.

Exclusion criteria were:

Seizure without fever.

Parent disaffiliates.

These people were divided into two groups with permuted-block randomization. The first group included 29 children receiving topiramate (Topiramate Daroupakhsh 50 mg Tab), 5-9 mg/kg/day, in 2 divided doses. The second group comprised 25 children receiving oral diazepam (Diazepam Loghman 2 mg Tab), 1 mg/kg/day, in the first three days with fever episodes.

Before the initiation of preventive treatment, necessary recommendations were given to the parents and their informed consent was completed. 

All patients received monthly follow-up calls and were examined by a pediatric neurologist every two months. All topiramate subjects underwent a sonography examination by the end of the course. All patients were followed for early prognosis during first year (Seizure currency and the side effects of drugs). 

Data were analyzed with Chi-square and t-test, using SPSS 20 (Chicago, IL, USA). 

## Results

**Figure 1:**
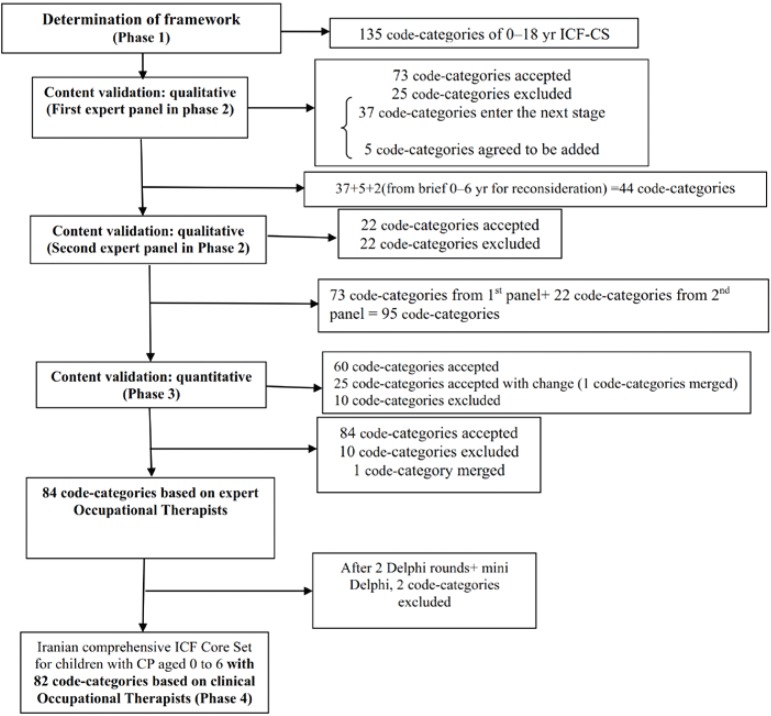
Flow diagram for trial (based on CONSORT 2010 flow diagram

Thirteen out of 54-research subjects were excluded (3 for a change in initial diagnosis, and 10 for medication withdrawal). Administration of topiramate was discontinued for 7 patients (4 cases for developing skin rashes, 2 cases for developing narcosis, and 1 case for developing fever and irritability). Although the first 4 cases had mild skin rashes, medication was discontinued due to their fear of intensifying symptoms. In two other cases, withdraw was because of parents’ anxiety. Thirty-four patients completed the one-year treatment course (17 patients in the topiramate group and 17 patients in the diazepam group) (Figure 1).

All patients had a previous history of taking phenobarbital but discontinued for its side effects (67.7% for skin allergies and 32.4% for irritability). 

Among the patients, 10 subjects were female and 24 subjects were male. In terms of age, 61.8% of subjects were older than 12 months and 38.2% were 12-month old or younger. In addition, 67.6% and 32.4% of subjects developed simple febrile seizure and complex febrile seizure, respectively. 

The two groups were compared in terms of age, sex, type of seizure, number of recurrent risk factors (criteria for receiving preventive therapy), and family history positive for epilepsy, fever, and seizure. There was no significant between-group difference in these factors (P>0.05) (Table 1).

Seizure recurrence was not observed in the patients in the following year. Certainly, the two groups would not be different in the rate of recurrent febrile seizure.

**Figure 2:**
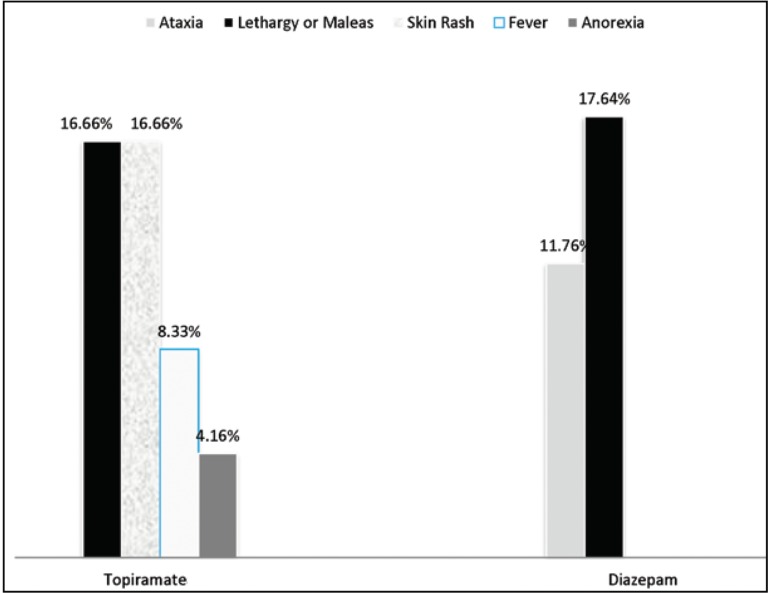
Side effects of drugs in both diazepam and topiramate groups

In the diazepam group, 3 subjects developed significant drowsiness and 2 subjects developed balance disorder (29.4% in total). In the topiramate group, 4 cases developed mild skin rashes, 4 subjects developed irritability, 2 subjects had fever, and 1 subject developed anorexia (45.8% in total) (P=0.008). In general, a major complication was not observed in any of the patients (Figure 2).

## Discussion

In our study, none of the patients receiving preventive therapy with diazepam developed recurrent febrile seizure. The effectiveness of taking diazepam during fever episodes for controlling febrile seizure is reported (3). The lack of difference between the outcomes of diazepam and topiramate therapies in our study may indicate the effectiveness of topiramate in preventing the incidence of febrile seizure. Topiramate could be used for the prevention of recurrent febrile seizure owing to its protective effect on blood-brain barrier (16). Children with probable risk of recurrent febrile seizure or epilepsy received monotherapy with topiramate and a 10-yr follow-up, during which no seizure recurrence was reported. The early use of topiramate (as an antiepilepsy drug) was very effective for children at risk of recurrent febrile seizure (15). The protective effect of topiramate on neurons) could prevent the development of hippocampal sclerosis and symptomatic epilepsy in children with recurrent febrile seizure (17). The prevalence of 10% to 20% was reported for recurrent febrile seizure in children receiving diazepam during fever episodes (9, 10, 19-23). The non-recurrence of febrile seizure in these patients can be attributed to low activation threshold, small sample size, and one-year follow-up period. On the other hand, the subjects in our study had at least 2 to 5 risk factors for recurrent febrile seizure (with 2.76, on average, risk factors for recurrent seizure in diazepam group), non-recurrent seizure in diazepam group can be attributed to its preventive effectiveness. The number of risk factors for febrile seizure recurrence in the topiramate group was 2.47, on average; the non-recurrence of febrile seizure in this group can also be attributed to its preventive effectiveness.

In our study, no serious complication was observed; whereas, one-third of diazepam group developed mild complications. These findings are consistent with previous studies reporting the prevalence of almost 9%-37% for diazepam complications (10, 22, 24-27). The rate of mild complications was higher in the topiramate group. Since many of our subjects were included for having a history of phenobarbital-induced skin allergy, this background can justify 4 cases with skin allergy in topiramate group. 

**Table 1:** Comparison of patients' specifications in diazepam and topiramate groups

**Variable**	**Treatment Group**		
	**Diazepam**	**Topiramate**	***P*** **-value**
Ratio of patients ≤ 12 months to patients >12 months	0.88	0.416	0.290
Ratio of boys to girls	2.4	2.4	1.00
Seizure type (ratio of simple to complex seizures)	1.83	2.4	0.714
Mean number of risk factors for recurrent seizure	2.76	2.74	0.301
Family history positive for epilepsy (ratio of people with and without family history)	21.42	21.42	1.00
Family history positive for fever and seizure (ratio of people with and without family history)	0.7	0.307	0.271

Although this study suggested diazepam as a medicine with minimum complications, implementation of it into normal population would probably result in even lower number of complications. Despite this, the prevalence of 11%-56% was reported for topiramate complications; whereas, some of these complications were observed in less than half of our subjects (28-31). 

**In conclusion**, despite having mild complications, topiramate can be used as an alternative medicine for children with a high risk of recurrent febrile seizure, when phenobarbital and diazepam cannot be used. For having fewer complications, daily dose of diazepam during fever episodes is a better choice; however, it is not adequately efficient in the early hours after the onset of fever and or for low-grade fevers. Further studies are recommended to compare topiramate with other medicines such as phenobarbital, using a larger sample size.

## References

[1] Chung S (2014). Febrile seizures. Korean J Pediatr.

[2] Swaiman KF, Ashwal S, Ferriero DM, Schor NF (2012). Swaiman's pediatric neurology: principles and practice.

[3] Steering Committee on Quality Improvement and Management, Subcommittee on Febrile Seizures. Febrile Seizures: Clinical Practice Guideline for the Long-term Management of the Child with Simple Febrile Seizures (2008). Pediatrics.

[4] Hirtz DG (1989). Generalized tonic-clonic and febrile seizures. Pediatric Clinics of North America.

[5] Shlomo Shinnar (2003). Febrile Seizures and Mesial Temporal Sclerosis. Epilepsy Curr.

[6] Rosman NP, Colton T, Labazzo J, Gilbert PL, Gardella NB, Kaye EM (1993). A controlled trial of diazepam administered during febrile illnesses to prevent recurrence of febrile seizures. N Engl J Med.

[7] Sugai K (2010). Current management of febrile seizuresin Japan: an overview. Brain Dev.

[8] Lux AL (2010). Treatment of febrile seizures: historical perspective, current opinions, and potential future directions. Brain Dev.

[9] Tonekaboni S, Beyraghi N, Barzegar M, Vesal A (2005). Comparison between Diazepam and Phenobarbital in prevention of febrile seizure. Iran J Pediatr.

[10] Darreh F, Kahbazi M (2008). Comparing the effect of intermittent Diazepam and continuous Phenobarbital in prophylaxis of recurrence of febrile seizure. AMUJ.

[11] Bernstein D, Shelov SP (2004). Pediatrics for medical students: Lippincott Williams & Wilkins; 2003. Epilepsy in children.

[12] Duffner P, Baumann R, Green J (2008). Febrile seizures: clinical practice guideline for the long-term management of the child with simple febrile seizures. American Academy of Pediatrics. Steering Committee on Quality Improvement and Management. Subcommittee of Febrile Seizures. Pediatrics.

[13] Kliegman RM, Stanton B, Geme JS, Schor NF, Behrman RE (2016). Nelson textbook of pediatrics.

[14] Shinnar S, Glauser TA (2002). Febrile seizures. J Child Neurol.

[15] Waruiru C, Appleton R (2004). Febrile seizures: an update. Arch Dis Child.

[16] Qiu P-L, Shi Y, Sun D-K, Wang Y (2011). Clinical and EEG features in children with febrile seizures after antiepileptic drug therapy. Chin J Contemp Pediatr.

[17] Sendrowski K, Sobaniec W, Sobaniec-Lotowska ME, Artemowicz B (2007). Topiramate as a neuroprotectant in the experimental model of febrile seizures. Therapeutic-nursing problems since born to old age.

[18] Lotowska JM, Sobaniec-Lotowska ME, Sendrowski K, Sobaniec W, Artemowicz B (2008). Ultrastructure of the blood-brain barrier of the gyrus hippocampal cortex in an experimental model of febrile seizures and with the use of a new generation antiepileptic drug-topiramate. Folia Neuropathologica.

[19] Knudsen FU, Vestermark S (1978). Prophylactic diazepam or phenobarbitone in febrile convulsions: a prospective, controlled study. Archdischild.

[20] Beyraghi N, Hatamian B, Vesal A, Tonekaboni S (2008). Comparison between diazepam and phenobarbital in prevention of febrile seizure: clinical trial. Iran J Child Neurol.

[21] Badv R, Aharchi B, Kamali K (2013). Nitrazepam Versus Diazepam as Intermittent Prophylaxis for Febrile Seizures. ZUMS Journal.

[22] Amouian S, JALILI AM, Arabi M (2014). Comparing Clobazam with Diazepam in Preventing Febrile Seizure in Children: A Randomized Clinical Trial. J Mazand Univ Med Sci.

[23] Autret E, Billard C, Bertrand P, Motte J, Pouplard F, Jonville AP (1990). Double-blind, randomized trial of diazepam versus placebo for prevention of recurrence of febrile seizures. J Pediatr.

[24] Hemmati M, Rezaei M (2012). The effect of different oral dose Diazepam on preventing seizure in Febrile Convulsion. J Kermanshah Univ Med Sci.

[25] Costa M, Silva EA, Silva AE, Guerreiro MM (1996). (Intermittent prophylaxis in febrile seizures with oral diazepam: study of 82 cases). Arq Neuropsiquiatr.

[26] Guerreiro MM, Costa M, Bellomo MA, Sabino SH, Silva EA, Scotoni AE (1992). Intermittent prophylaxis in febrile seizures with oral diazepam. Arquivos de Neuro-psiquiatria.

[27] Daugbjerg P, Brems M, Mai J, Ankerhus J, Knudsen F (1990). Intermittent prophylaxis infebrile convulsions: diazepam or valproic acid?. Acta Neurologica Scandinavica.

[28] Mula M, Trimble MR, Lhatoo SD, Sander JW (2003). Topiramate and psychiatric adverse events in patients with epilepsy. Epilepsia.

[29] Grosso S, Galimberti D, Farnetani M, Cioni M, Mostardini R, Vivarelli R (2005). Efficacy and safety of topiramate in infants according to epilepsy syndromes. Seizure.

[30] Al Ajlouni S, Shorman A, Daoud A (2005). The efficacy and side effects of topiramate on refractory epilepsy in infants and young children: a multi-center clinical trial. Seizure.

[31] Giannakodimos ST, Georgiadis G, Tsounis ST, Triantafillou N, Kimiskidis V, Giatas K (2005). Add-on topiramate in the treatment of refractory partial-onset epilepsy: clinical experience of outpatient epilepsy clinics from 11 general hospitals. Seizure.

